# Acid evoked thermal hyperalgesia involves peripheral P2Y1 receptor mediated TRPV1 phosphorylation in a rodent model of thrombus induced ischemic pain

**DOI:** 10.1186/1744-8069-10-2

**Published:** 2014-01-09

**Authors:** Soon-Gu Kwon, Dae-Hyun Roh, Seo-Yeon Yoon, Ji-Young Moon, Sheu-Ran Choi, Hoon-Seong Choi, Suk-Yun Kang, Ho-Jae Han, Alvin J Beitz, Seog Bae Oh, Jang-Hern Lee

**Affiliations:** 1 Department of Veterinary Physiology, College of Veterinary Medicine and Research Institute for Veterinary Science, Seoul National University, Seoul 151-742, Republic of Korea; 2 Department of Maxillofacial Tissue Regeneration, School of Dentistry, Kyung Hee University, Seoul 130-701, Republic of Korea; 3 Laboratory of Molecular Signal Transduction, Center for Neural Science, Korea Institute of Science and Technology (KIST), Seoul 136-791, Republic of Korea; 4 Acupuncture, Moxibustion & Meridian Research Group, Medical Research Division, Korea Institute of Oriental Medicine, Daejeon, Republic of Korea; 5 Department of Veterinary and Biomedical Sciences, College of Veterinary Medicine, University of Minnesota, St Paul, MN 55108, USA; 6 Pain Cognitive Function Research Center, Dental Research Institute and Department of Neurobiology and Physiology, Department of Brain and cognitive Sciences, Seoul National University, Seoul 110-749, South Korea

**Keywords:** Ischemic pain, Acid, ATP, Thermal hyperalgesia, P2Y1 receptor, TRPV1, Phosphorylation

## Abstract

**Background:**

We previously developed a thrombus-induced ischemic pain (TIIP) animal model, which was characterized by chronic bilateral mechanical allodynia without thermal hyperalgesia (TH). On the other hand we had shown that intraplantar injection of acidic saline facilitated ATP-induced pain, which did result in the induction of TH in normal rats. Because acidic pH and increased ATP are closely associated with ischemic conditions, this study is designed to: (1) examine whether acidic saline injection into the hind paw causes the development of TH in TIIP, but not control, animals; and (2) determine which peripheral mechanisms are involved in the development of this TH.

**Results:**

Repeated intraplantar injection of pH 4.0 saline, but not pH 5.5 and 7.0 saline, for 3 days following TIIP surgery resulted in the development of TH. After pH 4.0 saline injections, protein levels of hypoxia inducible factor-1α (HIF-1α) and carbonic anhydrase II (CA II) were elevated in the plantar muscle indicating that acidic stimulation intensified ischemic insults with decreased tissue acidity. At the same time point, there were no changes in the expression of TRPV1 in hind paw skin, whereas a significant increase in TRPV1 phosphorylation (pTRPV1) was shown in acidic saline (pH 4.0) injected TIIP (AS-TIIP) animals. Moreover, intraplantar injection of chelerythrine (a PKC inhibitor) and AMG9810 (a TRPV1 antagonist) effectively alleviated the established TH. In order to investigate which proton- or ATP-sensing receptors contributed to the development of TH, amiloride (an ASICs blocker), AMG9810, TNP-ATP (a P2Xs antagonist) or MRS2179 (a P2Y1 antagonist) were pre-injected before the pH 4.0 saline. Only MRS2179 significantly prevented the induction of TH, and the increased pTRPV1 ratio was also blocked in MRS2179 injected animals.

**Conclusion:**

Collectively these data show that maintenance of an acidic environment in the ischemic hind paw of TIIP rats results in the phosphorylation of TRPV1 receptors via a PKC-dependent pathway, which leads to the development of TH mimicking what occurs in chronic ischemic patients with severe acidosis. More importantly, peripheral P2Y1 receptors play a pivotal role in this process, suggesting a novel peripheral mechanism underlying the development of TH in these patients.

## Introduction

During pathological conditions such as ischemia, inflammation and tissue injury, a huge array of endogenous chemicals are released into the tissue that contribute to sensory phenomena including pain and hyperalgesia. These endogenous pain producing and sensitizing substances activate nociceptive nerve endings by binding to receptor molecules on their surface. Increasing evidence indicates that different sets of molecules contribute to mechanical and thermal hypersensitivity via several mechanisms in peripheral tissue [1-4]. A representative molecule is TRPV1, which is a noxious heat stimulus sensor and its activation results in a decreased threshold for heat stimuli [5-7]. It is well recognized that activation of TRPV1 receptors by various pro-algesic substances such as protons, ATP, bradykinin and prostaglandins contribute to peripheral sensitization, particularly to heat stimuli during the pathological states [1,5].

Ischemic pain typically results from a shortage of blood supply to tissue and is associated with a number of pathological states including atherosclerosis and intermittent claudication resulting from peripheral arterial disease (PAD) [8,9]. Intermittent claudication is a type of muscle pain which occurs during ambulation, and this is an early symptom in PAD patients [10]. As the disease progresses, severe limb ischemia might result in pain at rest without exercise [10]. Although patients with PAD demonstrate a range of ischemic pain severity, with abnormal response to mechanical and heat stimulus [9,11,12], mechanistic differences in pain intensity and modality have been poorly explored. Since there were no proper animal models that mimic the mechanisms of peripheral ischemia observed in PAD patients, we previously developed and described a new animal model of thrombus-induced ischemic pain (TIIP) in the rat and investigated the peripheral mechanisms underlying this ischemia induced pain [13,14]. TIIP rats showed persistent bilateral mechanical allodynia and we demonstrated that peripheral acid-sensing ion channels (ASICs) and P2X receptors were involved in the maintenance of this thrombus-induced ischemic pain [14]. These results indicated that protons associated with low ischemic pH and an increased concentration of ATP at the ischemic site played crucial roles in the development of ischemia-induced peripheral sensitization.

Thermal hyperalgesia (TH) has been shown to develop in chronic ischemic patients with severe acidosis and thus we would anticipate that it should be present in the TIIP model. While ischemia-induced conditions are closely associated with TRPV1 activation [15-19], and TRPV1 is the endpoint target of a variety of sensitizing substances including protons and ATP [1,5,6], TIIP rats show no hypersensitivity to heat stimuli, i.e. thermal hyperalgesia (TH), and changes in the pharmacological activity of peripheral TRPV1 is not detected in this model [13,14]. These findings suggest that the lack of TH development in TIIP animals might be caused by the internal ischemic environment, such that the acidity and the ATP concentration are inadequate to activate TRPV1. Previously, we have demonstrated a facilitatory effect of acidic pH on ATP-induced hypersensitivity in the normal rat’s hind paw utilizing α,βme-ATP as a substitute for endogenous ATP [20]. Although injection of acidic pH or α,βme-ATP alone failed to activate TRPV1 and P2Y1 receptors and produce TH, a combination of acidic pH in the presence of α,βme-ATP, did induce a transient TH via the activation of peripheral P2Y1 and TRPV1 receptors [20]; these results suggested that the synergistic action of acidic pH and ATP directly or indirectly activated TRPV1 and P2Y1 receptors. However, the interrelationship between these receptors and the potential molecular mechanisms underlying the development of TH under ischemic conditions is unclear. In the present study we used the TIIP animal model to investigate the peripheral induction mechanisms of TH shown in chronic ischemic patients with severe acidosis. In this regard, the present study examines: (1) whether injection of acidic saline into the hind paw causes the development of TH and changes the local tissue ischemic and acidic conditions in TIIP rats; (2) which proton and ATP sensing receptors are involved in this newly developed TH; and finally (3) whether there are functional interactions among these TH related receptors.

## Materials and methods

### Animals

Sprague–Dawley rats (weighing 350 to 400 g) were obtained from the Laboratory Animal Center of Seoul National University (SNU). All experimental procedures were approved by the SNU Animal care and Use Committee and performed according to guidelines established by NIH (NIH Revised Guide for the Care and Use of Laboratory Animals; eighth edition, National Academies Press, 2011). Animals were housed in a standard animal facility maintained in a 12 h light/dark cycle and a constant room temperature (maintained between 20–25°C) with 40–60% humidity. Food and water were available *ad libitum* throughout the investigation.

### TIIP surgery

TIIP surgery was performed according to the method described by Seo *et al*. [13]. Briefly, rats were first anesthetized intraperitoneally with a Zoletil 50® (Virbac laboratories, 06516 Carros) and Rompun® (Bayer Korea Ltd, Ansan) mixture (combining 2.5 mg of Zoletil 50® with 0.47 mg of Rompun® in saline), and then a small incision was made in the femoral triangle of the left hindlimb. The femoral artery was separated from the femoral vein and nerve by a piece of moisture-resistant film (Parafilm®, Chicago, IL, USA, 1 × 2.5 cm) to prevent possible ferrous chloride damage to these structures. A filter paper disc (0.5 × 0.5 cm; No. 2, Toyo Roshi Kaisha, Ltd., Japan) soaked with 20% FeCl_2_ (FeCl_2_ · 4H_2_O, Sigma, St. Louis, MO, USA) solution was placed on the femoral artery for 20 minutes. After the incision was surgically closed and covered by surgical dressing, animals were kept in a warming chamber (28°C) until they completely recovered from the anesthesia.

### Intraplantar drug administration and experimental procedures

**Figure 1:**
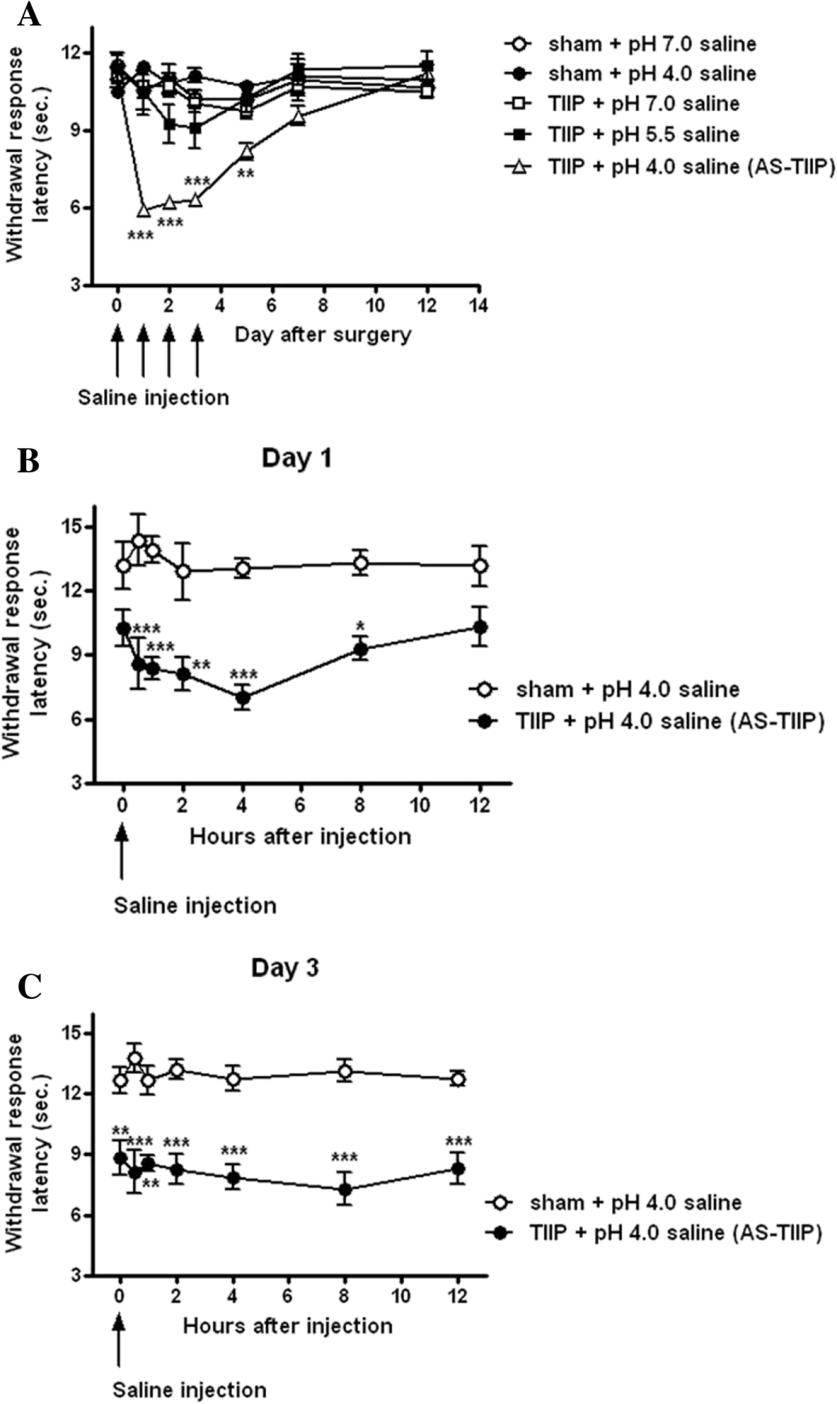
**Thermal hyperalgesia (TH) caused by intraplantar injection of acid in thrombus-induced ischemic pain (TIIP) rats. (A)** Long-term effects of acid (D0-3 post-surgery) induced TH in TIIP rats. We evaluated TH of sham control rats (pH 7.0 and 4.0, n = 7 and 5 in each group) and TIIP rats (pH 7.0, 5.5 and 4.0, n = 5, 5 and 9 in each group) at day 1, 2, 3, 5, 7 and 12 after the surgery. TH was measured 2 hours after saline injection to avoid its acute effects. Only in the TIIP + pH 4.0 saline (acidic saline injected TIIP, AS-TIIP) group, did significant TH develop beginning on day 1 after the injection of pH 4.0 saline and continuing for the duration of the injection period (**P < 0.01, ***P < 0.001 vs sham + pH 7.0 saline). **(B)** Short-term effects of acid-induced TH in TIIP rats at day 1 post-surgery. We evaluated TH in sham and TIIP rats following injection of pH 4.0 saline (n = 6 in each group) at 30 min, 1, 2, 4, 8 and 12 hours after pH 4.0 saline injection. After pH 4.0 saline injection, AS-TIIP rats showed prominent TH compared to sham rats; TH was observed from 30 min to 8 hours post-injection (*P < 0.05, **P < 0.01 and ***P < 0.005 vs sham + pH 4.0 saline). **(C)** Short-term effects of acid-induced TH in TIIP rats at day 3 post-surgery. We evaluated TH in sham and TIIP rats following injection of pH 4.0 (n = 6 in each group) at 30 min, 1, 2, 4, 8 and 12 hours after pH acid injection. Significant TH was present in AS-TIIP rats prior to pH 4.0 saline injections. Following pH 4.0 saline injections, AS-TIIP rats showed sustained TH, which did not recover until 12 hours post-injection (**P < 0.01, ***P < 0.001 vs sham + pH 4.0 saline).

After rats were briefly anesthetized with Gerolan®, inhalation anesthetic (Choong-wae, Hwa-seong, Kyonggi, Korea) in an acylic chamber, 30 μl of each drug or 50 μl of acidic saline was injected into the central sole region of the hind paw. The pH was adjusted with 1 N HCl solution [20,21]. In order to provide a more acidic environment to the ischemic hind paw, pH 4.0 saline was injected intraplantarly once daily from day 0 to 3 following TIIP surgery (acidic saline injected TIIP, AS-TIIP). The first injection of pH 4.0 saline was performed immediately after the surgery (day 0), and nociceptive behavioral testing was started beginning on day 1 post-surgery. Acid-induced thermal hyperalgesia (TH) was measured in two parts. The long-term effects of acid-induced TH were evaluated at days 1, 2, 3, 5, 7 and 12 after TIIP surgery. In order to avoid possible acute effects of acidic stimulation, during our examination of the long-term effects of acidic saline, we first examined TH beginning 2 hours after pH 4.0 saline injections (Figure 1A). In attempt to examine the possible short-term effects of acidic saline, we measured withdrawal response latency (sec) to heat over a 12-hour period following pH 4.0 injection on days 1 and 3 post-surgery. In this set of experiments we analyzed behavioral changes at 30 min, 1, 2, 4, 8 and 12 hours following the injection of pH 4.0 saline.

AMG9810 (a TRPV1 antagonist), chelerythrine (a PKC inhibitor), Amiloride (an ASICs blocker), TNP-ATP (a P2X receptor antagonist), or MRS2179 (a P2Y1 receptor antagonist) was injected into the hind paw in order to investigate the mechanisms underlying the development of thermal hyperalgesia (TH). The potential involvement of TRPV1 and PKC dependent pathways during the maintenance phase of TH was evaluated by a single injection of AMG9810 and chelerythrine at day 3 post-surgery. To further explore the intrinsic mechanisms underlying the development of TH, Amiloride, AMG9810, TNP-ATP and/or MRS2179 were repeatedly injected once a day (D0-D3 after surgery) 30 min before the acidic saline injection. Each control group received an intraplantar injection of the appropriate vehicle for each drug. Animals were randomly assigned to experimental groups and subsequent drug treatment and behavioral analyses were performed blindly.

### Assessment of thermal hyperalgesia

To assess nociceptive responses to noxious heat stimuli, we measured the paw withdrawal response latency (PWL) using a plantar test apparatus (Series 8, Model 390, IITC Life Science Inc., Woodland Hills, CA, USA), and evaluated animal’s sensitivity to noxious heat stimulation (thermal hyperalgesia). Before the test, rats were placed in a plastic chamber with a glass floor and allowed to acclimate for 15 minutes. Room temperature was constantly maintained between 26–28°C. A radiant heat source was positioned under the glass floor beneath the hind paw to be tested and withdrawal latency was measured by using a photoelectric cell connected to a digital clock. Baseline latency responses (10–15 sec) were determined before experimental treatment. The test was repeated in each hind paw at each time point and the mean withdrawal latency was calculated. A cut-off time of 20 seconds was used to protect the animal from excessive tissue damage.

### Resiniferatoxin treatment

In order to examine the possible role of TRPV1 containing peripheral nerve fibers, we treated the animals with the potent capsaicin analog, Resiniferatoxin (RTX, 0.3 mg/kg; Sigma, St. Louis, MO) dissolved in a mixture of 10% Tween 80, 10% ethanol, 80% normal saline [22]. After rats were anesthetized with 3% isoflurane in a mixture of N_2_O/O_2_ gas, either RTX or vehicle was injected subcutaneously in a volume of 0.2 ml into the scruff of the neck. The eye wipe reflex test was subsequently performed to check whether RTX treatment destroyed capsaicin sensitive primary afferent fibers at day 2 post-RTX injection. A diluted capsaicin solution (0.01%, dissolved in saline) was dropped onto cornea, and then the number and the duration of eye wipes were counted for 1 min. Following the eye wipe reflex test, we performed sham and TIIP surgery in vehicle-treated animals and in RTX-treated animals that showed no response to diluted capsaicin solution.

### Western blot analysis

Plantar muscle and skin from the ipsilateral hind paw were collected from anesthetized rats at postoperative day 3; to avoid acute effects of adjusted saline injection, we obtain tissue samples for western blot analysis at least 2 hours after saline injections. The tissue were homogenized in buffer containing 1 M Tris (pH 7.5), 1% NP-40, 0.5 M EDTA (pH 7.5), 50 mM EGTA, 1 M dithiothreitol, 1 M benzanidine and 0.1 M PMSF. The total amount of protein in each sample was determined using the Bradford dye assay prior to loading on polyacrylamide gels. The tissue homogenates (30 μg protein) were separated by 10% SDS-polyacrylamide gel electrophoresis and transferred to nitrocellulose. After the blots had been washed with TBST [10 mM Tris–HCl (pH 7.6), 150 mM NaCl, 0.05% Tween-20], the membranes were blocked with 5% skim milk for 1 hour. The muscle samples were incubated with primary antibodies for hypoxia inducible factor-1α (HIF-1α, 1:1000) or carbonic anhydrase II (CA II, 1:1000), which were purchased from the Santa Cruz Biotechnology (Delaware, CA). We utilized a TRPV1 polyclonal antibody (1:1000, Santa Cruz Biotechnology Inc., Santa Cruz, California, USA) and a pTRPV1 (phosphor S800) polyclonal antibody (1:1000, Abnova corporation, Taipei City, Taiwan) for skin samples. β-actin antibody was used as a loading control (Sigma, St. Louis, MO). After the secondary antibody reaction, the bands were visualized with enhanced chemiluminescence (Amersham Pharmacia Biotech, England, UK). The positive pixel area of specific bands was measured with a computer-assisted image analysis system (Metamorph®, version 6.3r2, Molecular Devices Corporation, PA) and normalized against the corresponding β-actin loading control bands. Then the ratio of pTRPV1 /TRPV1 expression was calculated. The mean values of the TRPV1 and pTRPV1/TRPV1 positive pixel area in the sham group was set at 100% and used for comparison with the experimental group.

### Immunohistochemistry

Animals were deeply anesthetized with an intra-peritoneal injection of a Zoletil-Rompun-saline mixture (2:1:2). Animals were perfused transcardially with calcium-free Tyrode’s solution, followed by a fixative containing 4% paraformaldehyde in 0.1 M phosphate buffer (pH 7.4). The ipsilateral DRGs (L4-L5) were collected after perfusion, post-fixed in the identical fixative for 4 hours and then placed in 30% sucrose in PBS (pH 7.4) at 4°C overnight. Frozen serial frontal sections (10 μm) were cut through the DRG L4–L5 using a cryostat (Microm, Walldorf, Germany). These serial sections were pre-blocked with 3% normal donkey serum and 0.3% Triton X-100 in PBS for 1 hour at room temperature. Tissue sections were incubated at 4°C with goat polyclonal TRPV1 antibody (1:250, Santa Cruz Biotechnology Inc., Santa Cruz, California, USA) for 48 h and followed by AlexaFluor 488 conjugated secondary antibody (1:500, invitrogen, Carlsbad, California, USA) for 2 hr at room temperature.

### Drugs

3,5-Diamino-N-(aminoiminomethyl)-6-chloropyrazinecarboxamide hydrochloride (Amiloride), (2E)-N-(2,3-Dihydro-1,4-benzodioxin-6-yl)-3-[4-(1,1-dimethylethyl)phenyl]-2-propenamide (AMG9810), Chelerythrine, 2′,3′–O–(2,4,6-Trinitrophenyl)adenosine-5′-triphosphate triethylammonium salt (TNP-ATP), 2′-Deoxy-N6-methyladenosine 3′,5′-bisphosphate tetrasodium salt (MRS2179) and [[(1R,2R,3S,4R,5S)-4-[6-Amino-2-(methylthio)-9H-purin-9-yl]-2,3-dihydroxybicyclo[3.1.0]hex-1-yl]methyl] diphosphoric acid mono ester trisodium salt (MRS2365) were purchased from Tocris (Ellisville, MO, USA). Amiloride, TNP-ATP, MRS2179 were dissolved in saline, respectively. AMG9810 was dissolved in 10% ethyl alcohol. Chelerythrine was dissolved in 5% DMSO. A 30 μl volume of one of the above drugs was injected intraplantarly for individual experiments.

### Statistical analysis

Statistical analysis was performed using Prism 5.1 (GraphPad Software, San Diego, CA). One-way analysis of variance (ANOVA) was performed to determine the change in TRPV1 and pTRPV1/TRPV1 concentrations in the ischemic hind paw skin. For posthoc analysis, the Newman-Keuls multiple comparison test was subsequently performed to determine significant differences among groups. A value of P < 0.05 was considered to be statistically significant. Behavioral data were tested using a two-way ANOVA to determine the overall effect of the drugs in each time-course.

## Results

### Long-term effects of intraplantar acidic saline injection on thermal hyperalgesia (TH) in TIIP rats

We injected pH adjusted saline solution (pH 4.0, 5.5, or 7.0) into the hind paw of TIIP rats once per day from days 0 to 3 after TIIP surgery to determine the potential effect of a more acidic, ischemic environment on thermal nociception. Repeated injection of pH 4.0 saline (Days 0 to 3 after surgery) caused a significant reduction in paw withdrawal latency to thermal stimuli in TIIP rats (Figure 1A). In order to investigate long-term effects of acid induced changes in heat hypersensitivity, we measured TH at day 1, 2, 3, 5, 7 and 12 after the surgery. The threshold for heat stimulation was evaluated at 2 hours post-injection in order to avoid potential acute effects of the pH 4.0 saline solution. TH developed on day 1 post-injection following TIIP surgery and was maintained throughout the entire 3-day injection period (Figure 1A; **P < 0.01, ***P < 0.005 vs sham + pH 7.0 saline), and gradually recovered over a period of several days after the injection period ended (Figure 1A). By contrast, the thermal threshold was unchanged in TIIP rats injected intraplantarly with pH 7.0 saline in the ipsilateral paw and thus, TH never developed. Importantly the two control groups (sham rats injected daily with pH 4.0 or pH 7.0 saline solutions) did not develop TH (Figure 1A) indicating that the ischemic condition was necessary for this acidic-induced TH to develop. Furthermore, one group of TIIP rats was injected into the ischemic hind paw with pH 5.5 saline on 3 consecutive days, but the injection of the pH 5.5 saline solution did not significantly decrease the threshold for the thermal stimulus (Figure 1A). Since only pH 4.0 saline induced the behavioral changes in thermal hypersensitivity in TIIP rats, we injected pH 4.0 saline into the ischemic hind paw in the remaining experiments as outlined below.

### Short-term effects of intraplantar acidic saline injection on thermal hyperalgesia (TH) in TIIP rats

In addition to the long-term changes in acid induced TH in TIIP rats, we observed changes in the time course of behavioral responses induced by a single injection of acidic saline (at days 1 and 3 post-surgery). After pH 4.0 injection, we observed changes in the withdrawal response latency (sec) at 30 min, 1, 2, 4, 8 and 12 hours post-injection (Figure 1B, 1C). On day 1, TIIP rats showed no significant TH prior to pH 4.0 injection compared to sham + pH 4.0 rats (Figure 1B). Following pH 4.0 saline injections, TIIP rats developed a significant TH compared to sham rats; TH was observed from 30 min to 8 hours post-injection. This thermal hyperalgesia slowly decreased beginning at 8 hours post-injection and the animals showed normal thermal responses by 12 hours after injection (Figure 1B; *P < 0.05, **P < 0.01 and ***P < 0.005 vs sham + pH 4.0 saline). At day 3 post-surgery, significant TH was already present in acidic saline injected TIIP (AS-TIIP) rats before pH 4.0 saline injection (Figure 1C; **P < 0.01 vs sham + pH 4.0 saline). Moreover, after pH 4.0 injections, AS-TIIP rats showed persistent TH and did not recover until 12 hours post-injection (Figure 1C; **P < 0.01 and ***P < 0.005 vs sham + pH 4.0 saline). Collectively, the TH induced by a single acidic saline injection was transient at day 1; however, following 2 more acidic saline injections, the TIIP rats developed persistent thermal hypersensitivity which was maintained for 2 days following the cessation of acidic saline injection (until day 5 post-surgery).

### Injection of acidic saline results in an increase in hypoxic status and acidity of the ischemic hind paw

**Figure 2:**
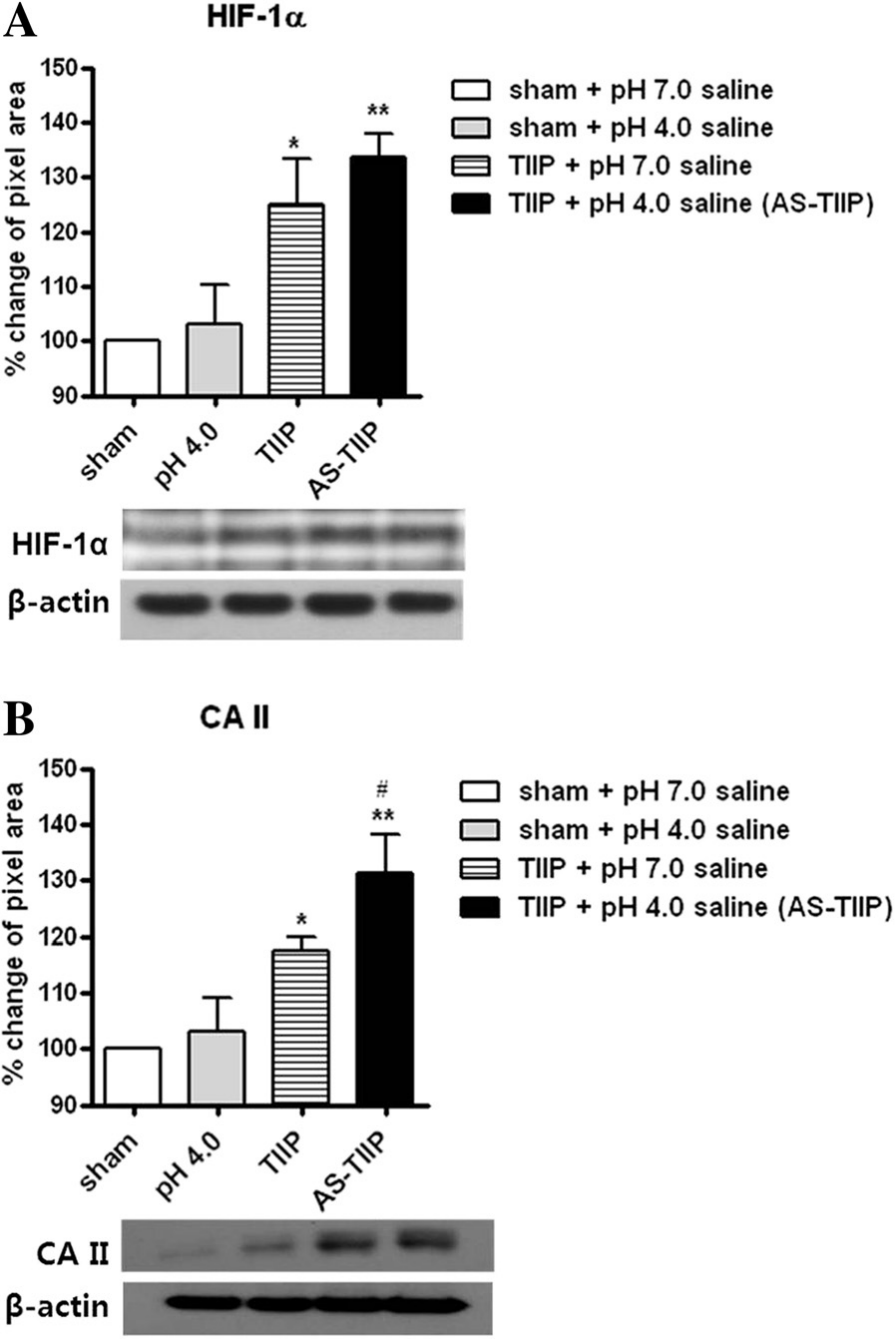
**Western blot analysis of hypoxia inducible factor-1α (HIF-1α) and carbonic anhydrase II (CA II).** HIF-1α (n = 5 in each group, day 3 post pH 4.0 saline injection) and, CA II (n = 4 in each group, day 3 post pH 4.0 saline injection) were quantitatively evaluated in sham and ischemic hind paw muscle lysates by western blotting. **(A)** The protein concentration of HIF-1α was significantly increased in the pH 7.0-treated thrombus induced ischemic pain (TIIP) group, and the protein concentration of HIF-1α was increased to an even greater extent in the pH 4.0 saline injected TIIP group (AS-TIIP); (*P < 0.05, **P < 0.01 vs sham + pH 7.0 saline). **(B)** The protein level of CA II was also significantly increased in pH 7.0-treated TIIP and AS-TIIP groups compared to the pH 7.0-treated sham group. pH 4.0 saline injection induced an additional increase in CA II expression in the AS-TIIP group compared to the pH 7.0-treated TIIP group. (*P < 0.05, **P < 0.01 vs sham + pH 7.0 saline and ^#^*P* < 0.05 vs TIIP + pH 7.0 saline). Data are presented as percentage of change (%) relative to sham control. β-actin was used as a loading control.

In order to evaluate whether repeated pH 4.0 saline injection induced changes in hypoxic status and acidity of peripheral hind paw tissue, hind paw muscle samples were collected at 3 days post-surgery following three days of acidic saline injection. To avoid potential acute effects of pH 4.0 and 7.0 saline injection, we euthanized the animals at least 2 hours post-injection. Since hypoxia inducible factor-1α (HIF-1α) is a well-known index of hypoxia and carbonic anhydrase II (CA II) has been reported to be a major indicator of pH imbalance [23-29], we examined changes in these two factors by western blot analysis (Figure 2). The protein concentration of HIF-1α was significantly increased in pH 7.0-treated TIIP rats, but there was a larger increase in acidic saline (pH 4.0) injected TIIP (AS-TIIP) rats (Figure 2A; *P < 0.05, **P < 0.01 vs sham + pH 7.0 saline). The protein concentration of CA II was also significantly increased in both the pH 7.0-treated TIIP and acidic saline (pH 4.0) injected TIIP animals compared to the pH 7.0-treated sham group (Figure 2B). The additional acidic saline injection resulted in significant increase in CA II expression in the AS-TIIP group compared to the pH 7.0-treated TIIP group (Figure 2B; *P < 0.05, **P < 0.01 vs sham + pH 7.0 saline, and # *P* < 0.05 vs TIIP + pH 7.0 saline). As a control group, we tested sham rats repeatedly injected with pH 4.0 saline, and there were no significant changes in either HIF-1α or CA II expression.

### TRPV1 and phosphorylated TRPV1 expression of hind paw lysates in acidic saline injected TIIP (AS-TIIP) rats

**Figure 3:**
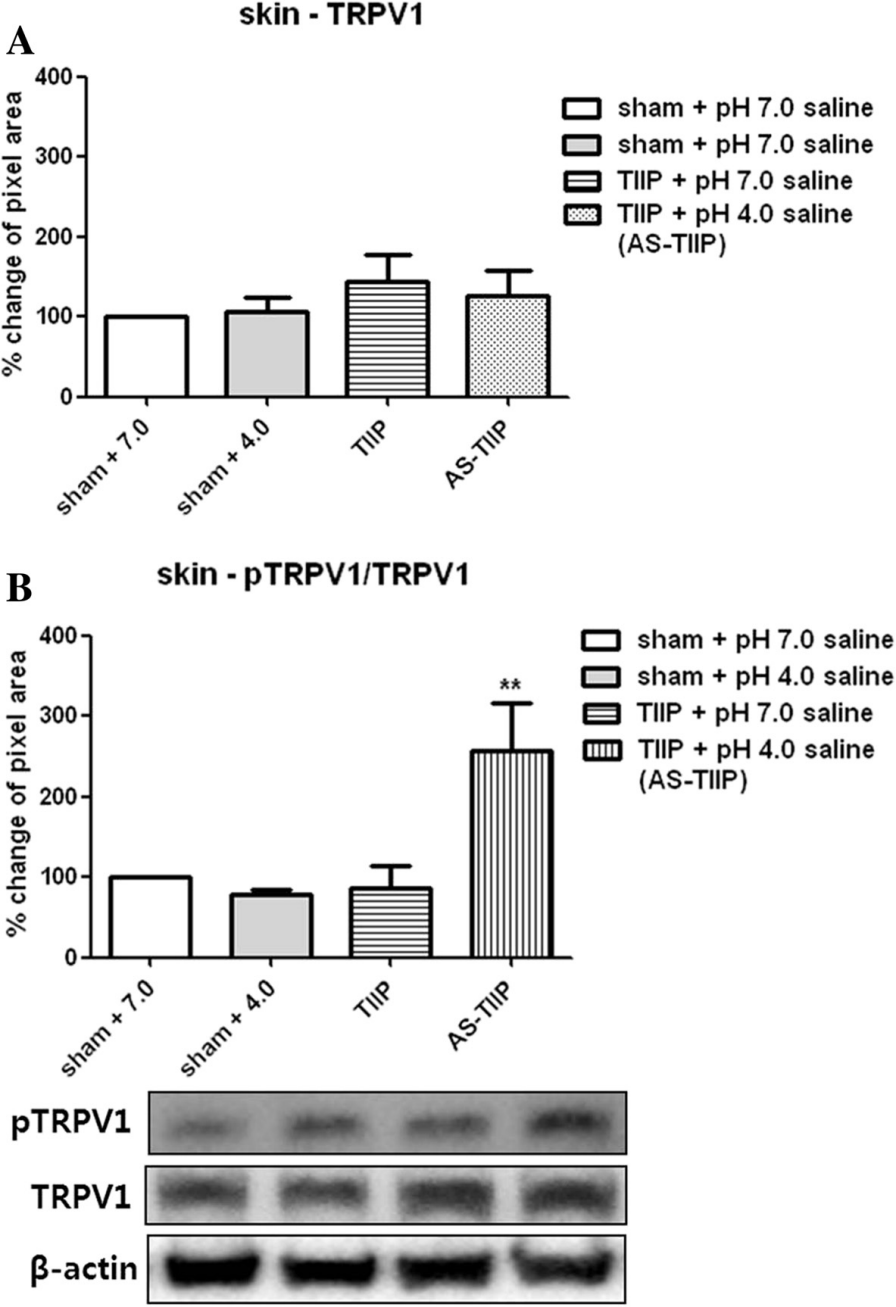
**Western blot analysis of TRPV1 and phosphorylated TRPV1 (pTRPV1) expression. (A)** TRPV1 receptor expression in hind paw skin lysates was quantitatively evaluated in sham + pH 7.0 saline, sham + pH 4.0, thrombus-induced ischemic pain (TIIP) + pH 7.0 saline, acidic saline (pH 4.0) injected TIIP (AS-TIIP) by western blotting. The protein expression of TRPV1 did not change in pH 7.0-treated TIIP and AS- TIIP rat groups compared to 7.0-treated sham groups (n = 4 in each group, 3 days after surgery and pH 4.0 injection). Each band was normalized against the corresponding β-actin band used as loading control. **(B)** We detected PKC dependent pTRPV1 using western blotting in the hind paw skin of sham + pH 7.0 saline, sham + pH 4.0 saline, TIIP + pH 7.0 saline and AS-TIIP (n = 4 in each group, 3 days after surgery and pH 4.0 saline injections). The expression of pTRPV levels significantly increased in AS-TIIP rats (**p < 0.01 vs sham + pH 7.0 saline) but not in pH 4.0 treated sham and pH 7.0-treated TIIP rats. The pTRPV1 level was normalized against corresponding total TRPV1. Data are presented here as a percentage change (%) compared to the sham control.

Since peripheral TRPV1 receptors were considered to be pivotal thermal sensors that contribute to the development of thermal hypersensitivity, we investigated possible changes in TRPV1 expression and phosphorylation rate in skin from the ischemic hind paw. To address this, we performed a western blot analysis of both TRPV1 and phosphorylated TRPV1 (pTRPV1) to determine whether peripheral ischemia alone or coupled with increased tissue acidity (induced by pH 4.0 saline injection) caused a change in TRPV1 expression and phosphorylation in paw lysates (Figure 3) at postoperative day 3 following three days of acidic saline injection. Expression of peripheral TRPV1 receptors was not significantly changed in pH 7.0-treated TIIP rats or in the acidic saline injected TIIP (AS-TIIP) group (Figure 3A). No statistical differences were detected among any of the 4 conditions at this time point. Quantitative analysis of TRPV1 expression was normalized against corresponding β-actin. TRPV1 has two phosphorylation sites for PKC-mediated phosphorylation: S502 and S800 [5]. The pTRPV1 antibody used in this study reacted with pTRPV1 at S800. Injection of acidic saline significantly increased the ratio of pTRPV1/TRPV1 and this was positively correlated with the induction of TH (Figure 3B; **P < 0.01 vs sham + pH 7.0 saline). Quantitative analysis of pTRPV1 expression was normalized against corresponding total TRPV1 expression.

### TRPV1 and phosphorylated TRPV1 expression of hind paw lysates in acidic saline injected TIIP (AS-TIIP) rats with resiniferatoxin (RTX) treatment

**Figure 4:**
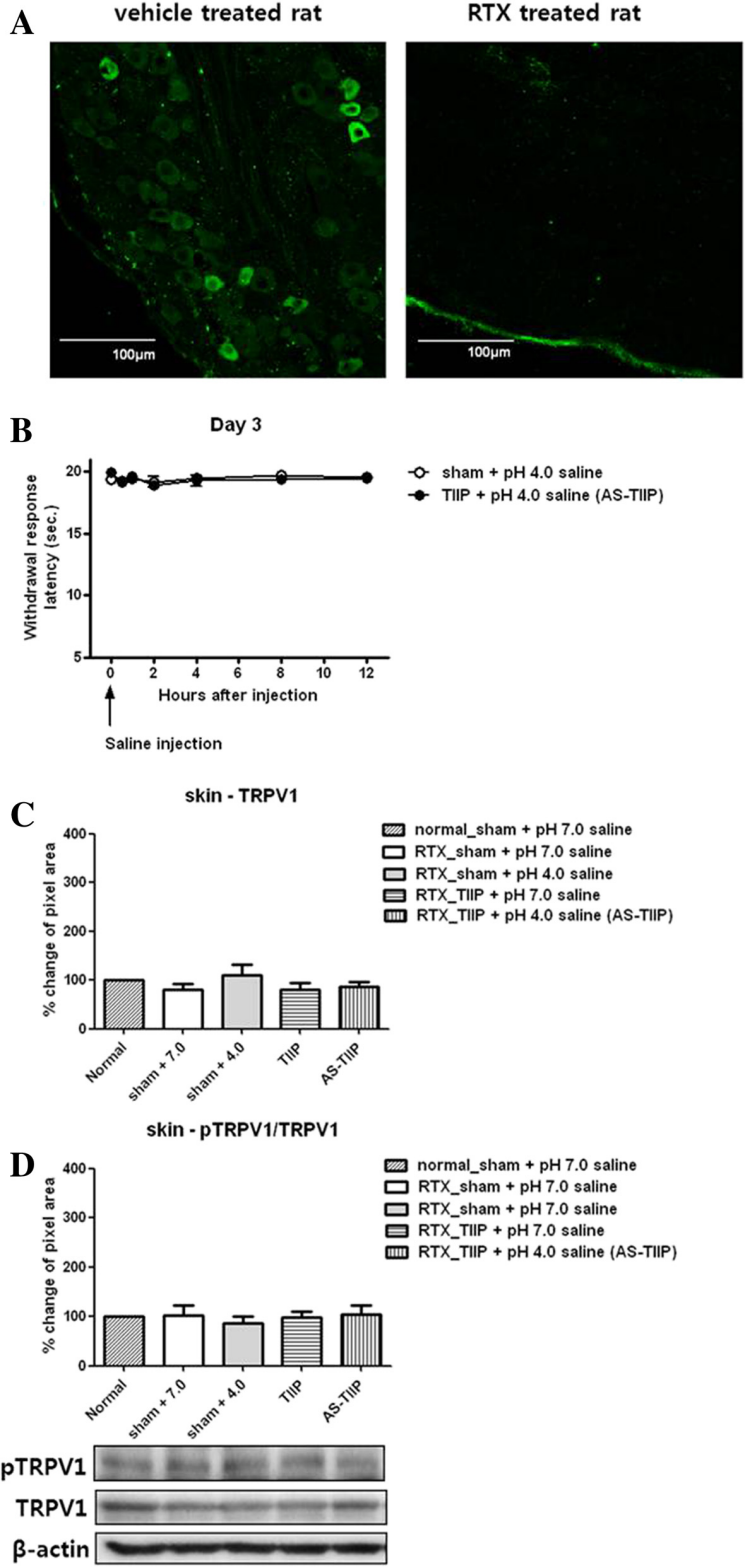
**The effect of acid in thermal hyperalgesia (TH) and TRPV1 expression in Resiniferatoxin (RTX) treated rats. (A)** Immunofluorescent images of rat DRG neurons. Thin sections (10 μm) of rat DRGs (L4-L5) were stained with antibodies against TRPV1 in the vehicle and RTX treated groups. The TRPV1-immunostaining in DRG neurons were almost completely abolished in RTX treated rats. Images are shown at 200× magnification. Scale bars represent 100 μm. **(B)** Short-term effects of acid induced TH in RTX treated TIIP rats at day 3. We evaluated the development of TH in RTX treated sham + pH 4.0 and acidic saline (pH 4.0) injected TIIP (AS-TIIP) rats (n = 4 in each group) at 30 min, 1, 2, 4, 8 and 12 hours after saline injection. RTX treated rats did not show TH following TIIP surgery and pH 4.0 injection. **(C)** TRPV1 receptor expression in hind paw skin lysates was quantitatively evaluated in RTX treated groups including sham + pH 7.0 saline, sham + pH 4.0 saline, TIIP + pH 7.0 saline and AS-TIIP by western blotting. The protein expression of TRPV1 did not change in any of the RTX treated groups compared to 7.0-treated sham groups (n = 6 in each group, 3 days after surgery and pH 4.0 injection). Each band was normalized against the corresponding β-actin band used as loading control. **(D)** pTRPV1 expression in hind paw skin lysates was quantitatively evaluated in RTX treated group including sham + pH 7.0 saline, sham + pH 4.0 saline, TIIP + pH 7.0 saline and AS-TIIP by western blotting. The protein expression of pTRPV1 did not change in any of the RTX treated groups compared to 7.0-treated sham groups (n = 6 in each group, 3 days after surgery and pH 4.0 injection). The pTRPV1 level was normalized against corresponding total TRPV1. Data are presented here as a percentage change (%) compared to the sham control.

Since TRPV1 is highly expressed in epidermal nerve fibers as well as in keratinocytes distributed in the epidermis of the skin [30-33], we perform an additional set of experiments to determine whether ischemic conditions result in the phosphorylation of TRPV1 receptors located in nerve terminals and/or epidermal keratinocytes. We treated separate groups of rats with the potent capsaicin analog Resiniferatoxin (RTX) two days before TIIP surgery to abolish capsaicin sensitive sensory neurons. Following RTX treatment, AS-TIIP rats did not show significant thermal hypersensitivity at day 3 (Figure 4B), and there were no detectable TRPV1 positive cells in lumbosacral DRGs indicating that TRPV1 positive neurons were completely abolished in these ganglia (Figure 4A). However, after RTX treatment, TRPV1 and pTRPV1 were still detected with western blotting; indicating that a significant portion of TRPV1 was found in non-neuronal sources compared to that in nerve endings (Figure 4C, 4D). Interestingly, although a large amount of TRPV1 still existed in RTX treated rats, there were no significant changes in pTRPV1/TRPV1 levels in AS-TIIP rats (Figure 4D). These results suggest that TRPV1 from non-neuronal cells does not contribute substantially to TH and that the up-regulation in pTRPV1 occurs predominantly in peripheral nerve fibers rather than keratinocytes.

### The involvement of TRPV1 and PKC dependent pathways in the maintenance of thermal hyperalgeisa (TH) in acidic saline injected TIIP (AS-TIIP) rats

**Figure 5:**
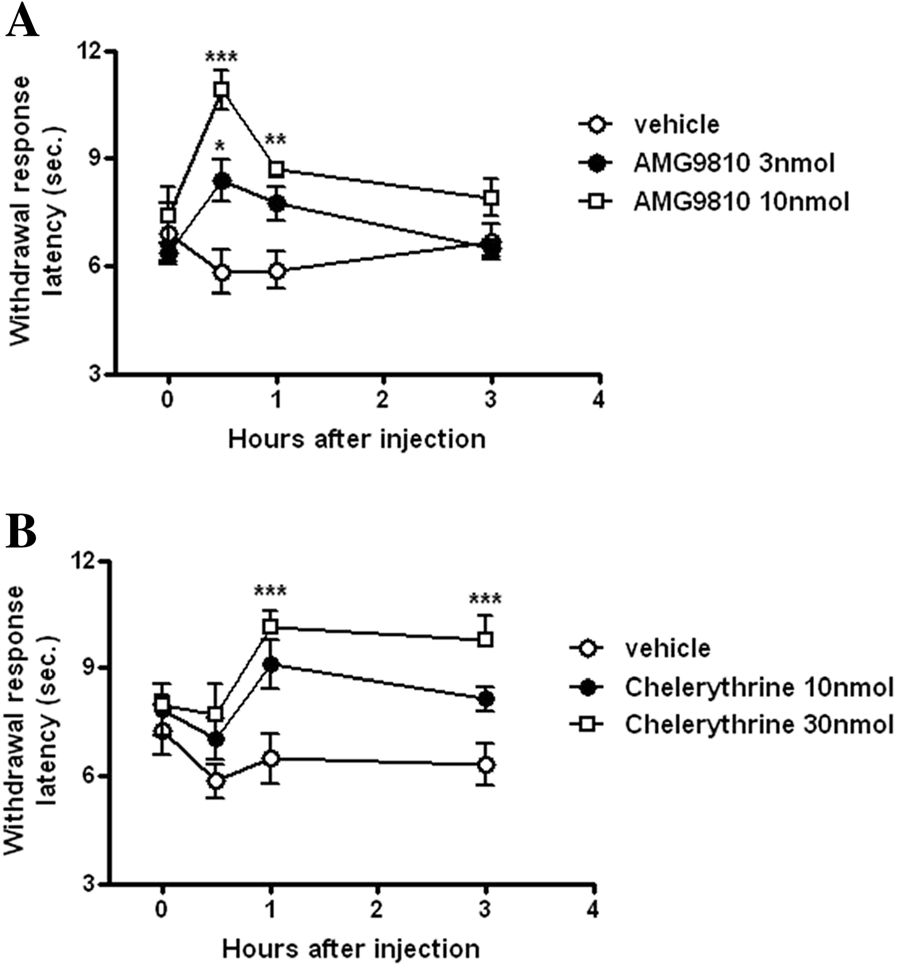
**The effect of AMG9810 and chelerythrine on established thermal hyperalgesia (TH) in acidic saline injected TIIP (AS-TIIP) rats.** Effects of AMG9810 (a TRPV1 antagonist) and chelerythrine (a PKC inhibitor) in established TH were evaluated at day 3 post-surgery and pH 4.0 injection. **(A)** Intraplantar injection of AMG9810 (3 and 10 nmol) was effective in reversing the established TH in a dose dependent manner (n = 4, 4, 5 in each group). The analgesic effect of AMG9810 was significant at 30 min and 1 h post-injection (*p < 0.05, **p < 0.01 and ***p < 0.001 vs vehicle). **(B)** Intraplantar injection of chelerythrine (10 and 30 nmol) dose dependently reversed TH at the 1 and 3 h time point after injection in established TH rats (n = 4, 6, 6 in each group, ***p < 0.001 vs vehicle).

To investigate the involvement of TRPV1 and PKC dependent signaling in this acid induced TH, the selective TRPV1 antagonist (AMG9810) or the PKC inhibitor (chelerythrine) were injected into the hind paw of AS-TIIP rats at day 3 post-surgery following daily pH 4.0 saline injection. (Figure 5). Following injection of AMG9810 (Figure 5A) or chelerythrine (Figure 5B) into the hind paw that had established TH, we examined possible behavioral changes in thermal hypersensitivity for 3 hours post-injection. Both AMG9010 (3 and 10 nmol) and Chelerythrine (10 and 30 nmol) dose-dependently reversed the established TH induced by acidic saline injection in TIIP rats. AMG9810 significantly reversed TH at 30 min and 1 h after injection, and chelerythrine inhibited TH at 1 h and 3 h after injection (Figure 5; *P < 0.05, **P < 0.01 and ***p < 0.001 vs vehicle).

### The involvement of proton sensing ion channels and P2 receptors in the induction of thermal hyperalgesia (TH) in acidic saline injected TIIP (AS-TIIP) rats

**Figure 6:**
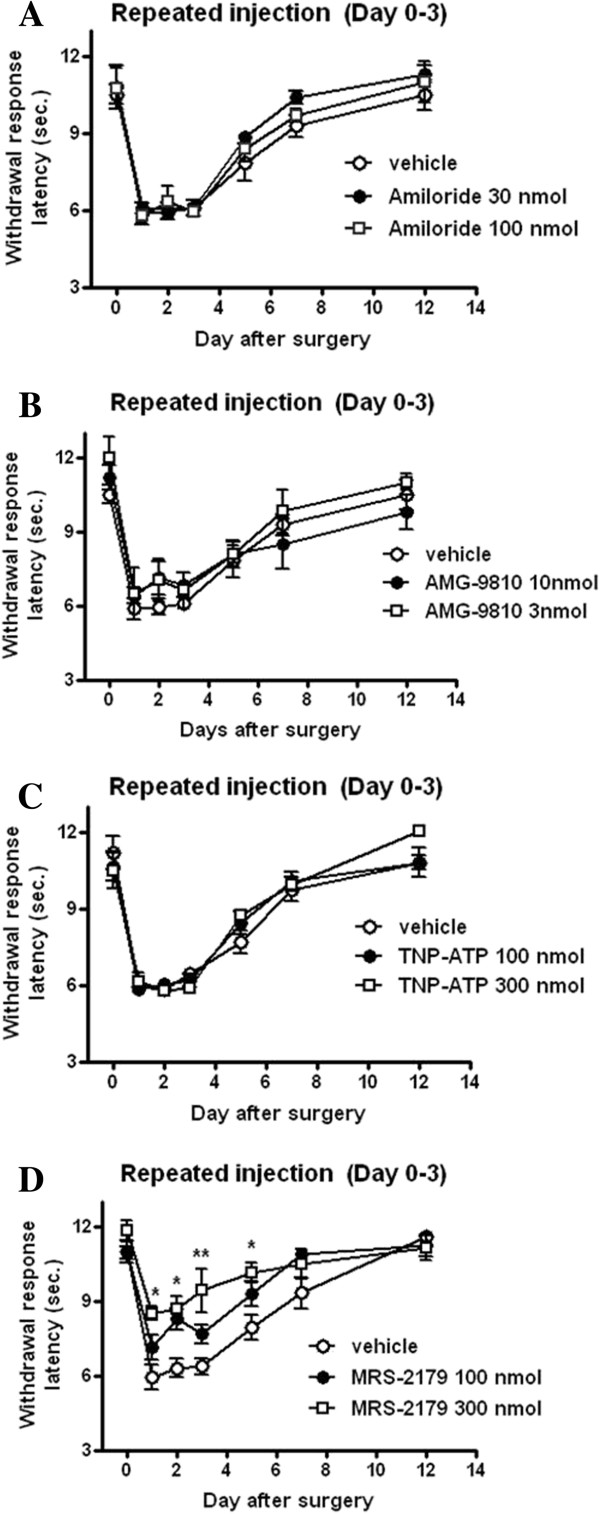
**The Effect of proton sensing ion channels and P2 receptors antagonists in the induction of thermal hyperalgesia (TH) in acidic saline injected TIIP (AS-TIIP) rats.** Graphs showing the effects of Amiloride (a ASICs blocker), AMG9810 (a TRPV1 antagonist), TNP-ATP (a P2X antagonist) and MRS2179 (a P2Y1 antagonist) on acidic injection-induced TH in TIIP rats. Each drug was repetitively injected to the hind paw 30 min before pH 4.0 saline injections (D0-3 post-surgery). Amiloride (**A**, 30 and 100 nmol), AMG9810 (**B**, 3 and 10 nmol) and TNP-ATP (**C**, 100 and 300 nmol) given before pH 4.0 saline injection did not prevent the development of TH (n = 7 in each group). Conversely, repetitive treatment with MRS2179 (**D**, 100 and 300 nmol, n = 4, 6, 5 in each group) effectively prevented the development of TH in the AS-TIIP group (*p < 0.05, **p < 0.01 vs vehicle).

To examine the potential role(s) of proton sensing ion channels and P2 receptors in the development of TH in the ischemic hind paw, we investigated the pharmacological effects of ASIC blockers and TRPV1, P2X and P2Y1 antagonists on TH in AS-TIIP rats. The potential role of proton-sensing receptors in the development of TH was evaluated first. To do this, rats were initially injected intraplantarly with either amiloride or AMG9810 daily 30 min before the pH 4.0 saline injections (Figure 6A, 6B, from days 0 to 3 after surgery). Both amiloride and AMG9810 were ineffective in preventing the development of TH in AS-TIIP rats (Figure 6A, 6B). It is interesting to note that the TRPV1 antagonist, AMG9810 fails to block the development of TH even though TRPV1 receptors appear to be involved in maintaining the lowered threshold for a noxious heat stimulus (Figures 5A and 6B). We next evaluated whether P2 receptors were involved in the development of TH caused by acidic saline injection under ischemic conditions (Figure 6C, 6D). To elucidate which P2 receptor subtype (P2X or P2Y1) contributed to induce TH, TIIP rats receiving pH 4.0 saline were pre-treated with a potent P2X antagonist, TNP-ATP (100 and 300 mol) or a selective P2Y1 antagonist, MRS2179 (100 and 300 nmol). Animals were pretreated with TNP-ATP or MRS2179 daily 30 min before the 4.0 saline injection (Figure 6C, 6D, from day 0 to 3). MRS2179 (100 and 300 nmol) dose-dependently blocked the development of TH, while TNP-ATP did not affect the onset of TH (Figure 6D *P < 0.05, **P < 0.01 vs vehicle).

### Peripheral P2Y1 receptors modulate TRPV1 activity by PKC-dependent phosphorylation in acidic saline injected TIIP (AS-TIIP) rats

**Figure 7:**
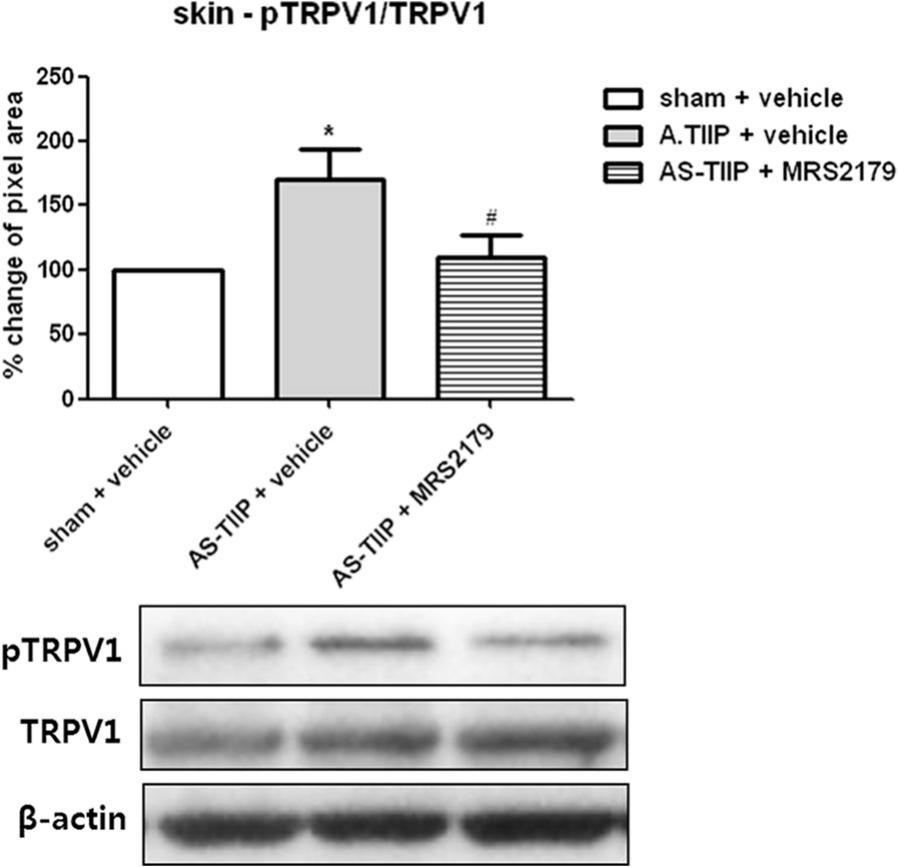
**The effect of MRS2179 on phosphorylated TRPV1 (pTRPV1) expression in acidic saline injected TIIP (AS-TIIP) rats.** TRPV1 and pTRPV1 were detected by using western blots in the hind paw skin of sham + vehicle, AS-TIIP + vehicle and AS-TIIP + MRS2179 (n = 6 in each group, 3 days after surgery and pH 4.0 saline injections). Vehicle and MRS2179 were repetitively injected into the hind paw 30 min before pH 4.0 injection. The level of pTRPV1 expression significantly increased in AS-TIIP rats, and repeated MRS2179 treatment decreased the ratio of pTRPV1 back to the sham control level (*p < 0.05 vs sham + vehicle and # p < 0.05 vs AS-TIIP + vehicle). The level of pTRPV1 expression was normalized against the corresponding total expression of TRPV1. Quantitative analysis of pTRPV1 was calculated as the percent (%) change with respect to the sham group.

Since P2Y1 receptors appeared to play a crucial role in acidic saline induced TH in TIIP rats (Figure 6D), an additional experiment was performed to test the hypothesis that P2Y1 receptors might modulate TRPV1 activity under ischemic conditions. To address this, we performed a western blot analysis of phosphorylated TRPV1 (pTRPV1) in the hind paw skin of sham + vehicle, AS-TIIP + vehicle and AS-TIIP + MRS2179 groups at postoperative day 3 following daily injections of pH 4.0 saline (Figure 7). Vehicle and MRS2179 were repetitively injected into the hind paw 30 min before pH 4.0 saline injection. Acidic saline injection into the hind paw of TIIP rats increased the expression of pTRPV1 (Figure 7, *P < 0.05 sham + vehicle). More importantly, repeated i.pl treatment with MRS2179 prior to the pH 4.0 saline injection returned the expression of pTRPV1 to baseline levels (Figure 7, # p < 0.05 vs AS-TIIP + vehicle). Quantitative analysis of pTRPV1 expression was normalized against corresponding total TRPV1 expression and calculated as a percent (%) change with respect to the sham group.

## Discussion

It has been reported that tissue acidity corresponds to the severity of hypoxic status, and the tissue acidic environment is a major factor for triggering ischemia related pathological consequences [23,34,35]. In terms of pain hypersensitivity, although low tissue pH at the ischemic site has been considered a crucial factor in the development of ischemic pain [8,14,36], the contribution of the acidic environment to ischemia-induced thermal hypersensitivity has not been delineated. The present study is the first to demonstrate that under peripheral ischemic conditions an increase in tissue acidity results in the development of thermal hyperalgesia (TH). Here, we injected pH 4.0 saline into the ischemic hind paw to evaluate how an acidic tissue environment affected both thermal hypersensitivity and TRPV1 activity under peripheral ischemic condition. Since hypoxia inducible factor-1α (HIF-1α) is a well-known indicator of hypoxia and carbonic anhydrase II (CA II) has been reported to be a major indicator of pH imbalance [23-29], we examine changes in these two factors in order to evaluate the degree of hypoxic injury and tissue acidity. We observed that repetitive acidic saline injection into the ischemic hind paw increased protein levels of both hypoxia inducible factor-1α (HIF-1a) and carbonic anhydrase II (CA II) in hind paw muscle (Figure 2). These results indicated that the presence of an acidic tissue environment intensified the ischemia-associated insults to the hind paw muscle tissue. Therefore, we postulate that injection of acidic saline into TIIP (AS-TIIP) rats represents a model of the ischemic condition with severe tissue acidosis.

Since the injection of pH 4.0 saline solution itself in sham rats did not affect thermal nociception (Figure 1), these findings indicated that an increased acidic environment in the presence of other pro-algesic substances under ischemic conditions could lead to the development of TH. It has been reported that there is a synergism between low tissue pH conditions and pro-algesic substances in their effect on nociceptor excitation [37,38]. In the skin-nerve preparation, a strong interaction between acidic pH and inflammatory mediators resulted in an increased prevalence and magnitude of nociceptor excitation [38]. This interaction was also shown in experimental tissue acidosis in human skin; e.g. the injection of additional inflammatory mediators into the acidic skin caused a more significant painful response [37]. In a previous study we demonstrated that the synergism between acidic saline and ATP produced a significant facilitation of nociception (mechanical allodynia and thermal hypersensitivity) in the normal rat hind paw [20]. Accordingly, it is reasonable to hypothesize that ATP may be one of the synergic factors that contribute to acidic pH-mediated pain development under ischemic conditions. Although we do not measure the exact concentration of tissue ATP in TIIP rats in the present study, it is well known that injured tissues possess a high concentration of extracellular ATP especially during early induction phase [39,40]. In TIIP rats, intraplantar injection of a P2 antagonist effectively alleviated mechanical allodynia at day 3 after surgery [13]. Moreover, in the current study, we injected acidic saline to the ischemic hind paw during this induction phase (D0 to D3), and observed increased thermal hypersensitivity. These results imply that during the early induction phase in which extracellular ATP concentration is elevated, injection of acidic saline could effectively modulate P2Y1 receptors, which could contribute to the development of increased thermal hypersensitivity.

Since previous studies have shown that P2Y1 and TRPV1 receptors are located in the same population of DRG neurons, we have examined the potential role of TRPV1 receptors in this process by examining the effect of injection of the specific TRPV1 antagonist AMG9810, using two different approaches. First, in order to investigate the potential involvement of TRPV1 in maintaining TH, we performed a single intraplantar injection of the selective TRPV1 antagoinst, AMG9810 at day 3. This injection resulted in short-term alleviation of the established TH (Figure 5A). Second, we performed repetitive intraplantar injection to further explore whether each acidic stimulation directly affected TRPV1 or not. AMG9810 were injected once a day (D0-D3 after surgery) 30 min before the acidic saline injection. Interestingly, this pre-blockade of TRPV1 before acidic saline failed prevent the induction of TH (Figure 6B). These results implied that TRPV1 was a final gate for acid induced TH in TIIP rats, but acid stimulation did not directly activate this channel.

In the current study, we discovered that peripheral P2Y1 receptors played a crucial role in acid induced TH, based on the fact that repeated pre-injection of MRS2179, a selective P2Y1 antagonist, effectively prevented the acidic saline-induced TH in AS-TIIP rats (Figure 6D). These results indicate that the acidic environment created by pH 4.0 saline injection in the ischemic hind paw modulates P2Y1 receptors, which in turn contribute to the development of TH. On the other hand, TRPV1 receptors do not appear to play a crucial role in the onset of TH in this model. In addition, we also concluded that ASICs did not contribute to thermal hypersensitivity in AS-TIIP rats (Figure 6A). Previously, we reported that peripheral ASICs were involved in maintaining ischemia-induced mechanical allodynia in TIIP [13]. However, we were unable to investigate their contribution to TH in the present study, since TIIP rats have normal heat sensitivity. In the current study, we observed that low tissue pH in conjunction with ischemia resulted in the development of TH in TIIP rats, and ASICs were not involved in this ischemic thermal hypersensitivity. On the other hand, ASICs did contribute to mechanical allodynia in AS-TIIP rats (data not shown). These results suggest that ASICs are a modality specific contributor to mechanical allodynia in acid-induced nociception, and there are several reports supporting the specific role of ASICs in mechanical sensing and hypersensitivity [20,41,42].

Although pH 4.0 is quite an extreme pH not often seen even in pathophysiological conditions, several studies have examined the actual pH level in the plantar tissue, and they demonstrated that the measured tissue pH is considerably higher than solution pH level before injection [21,41]. For example, Sluka *et al*. reported that the repeated injection of pH 4.0 saline into gastrocnemius muscle lowered the muscle pH averaged 6.5 with decreases in individual animals to pH 6 [21]. Therefore, we have assumed that a single injection of pH 4.0 saline would not extremely decrease the tissue pH, and further considered that temporary acidic pH tissue condition induced by pH 4.0 saline could mimic the pathophysiological state shown in peripheral ischemic condition. Collectively, the acidic environment is easily buffered by physiological buffering systems [15,21,43], it may be difficult to activate TRPV1 receptors directly, since they only respond under relatively low tissue pH (pH < 5–6) [44-46]. Therefore, there is the strong possibility that the interaction of the acidic environment and ATP indirectly activate TRPV1 via P2Y1 receptors which up-regulates intracellular signaling cascades, but this only occurs in an ischemic environment in which tissue acidosis is present.

It is well recognized that activation of TRPV1 receptors contributes to peripheral sensitization, particularly to heat stimuli. Pro-algesic substances such as protons, ATP, bradykinin and prostaglandins sensitize nociceptors either by directly modulating the sensitivity of membrane receptors or by up-regulating intracellular signaling cascades [5]. These signaling cascades include calcium dependent protein kinase (PKC), cyclic AMP-dependent protein kinase (PKA), and calcium-calmodulin dependent protein kinase (CaMKII) dependent phosphorylation [5]. Since PKC dependent modulation of TRPV1 had been reported to be the main pathway stimulated by inflammatory mediators [5,47,48], an analysis of phosphorylated TRPV1 that targeted S800 was performed in the present study. Sham (pH 7.0 and 4.0), TIIP and AS-TIIP rats did not show any alterations in TRPV1 expression (Figure 3A), whereas the ratio of pTRPV1/TRPV1 was significantly elevated in AS-TIIP rats (Figure 3B). Although it has been reported that PKC dependent TRPV1 phosphorylation is critical in the TRPV1 response to its agonist *in vitro*[7,49,50], this is the first evidence that increased phosphorylation of TRPV1 in peripheral tissues directly correlates with behavioral changes *in vivo.* Since TRPV1 is not only located in peripheral nerve fibers, but also in the keratinocytes distributed in the epidermis of the skin, our initial western blot data contain TRPV1 from both neuronal and non-neuronal origins. Therefore, in attempt to evaluate the contribution of non-neuronal TRPV1 to the up-regulation of the pTRPV1/TRPV1 ratio observed in AS-TIIP rats, we gave resiniferatoxin (RTX) to several groups of rats to destroy TRPV1 containing nerve fibers and subsequently examined the changes in the ratio of TRPV1 and pTRPV1 expression in paw lysates from sham (pH 7.0 and 4.0) and TIIP (pH 7.0 and 4.0) rats. Although TRPV1 was still present in hind paw lysates from RTX treated rats, there were no significant changes in the ratio of pTRPV1/TRPV1 in AS-TIIP rats (Figure 4D). These results indicated that TRPV1 receptors associated with keratinocytes or other non-neuronal sources did not contribute to the increase in pTRPV1/TRPV1 ratios that we observed in AS-TIIP animals. Collectively, these findings suggest that the transitional rate of PKC dependent phosphorylation of TRPV1 in peripheral nerve fibers is an important factor in the development of heat hypersensitivity in chronic ischemic conditions.

P2Y1 receptors are Gq-coupled receptors located in sensory neurons and these receptors have been implicated in sensory transduction [30,51,52]. There are several reports suggesting that there is a potential relationship between the P2Y1 and TRPV1 receptors that contributes to increased pain hypersensitivity [7,30,53-55]. P2Y1 receptors are located primarily in small diameter sensory neurons, and thus it is perhaps not surprising that over 80% of CGRP neuronal profiles in DRGs contain P2Y1 receptors [53]. Interestingly Gerevich *et al.* demonstrated that P2X_3_, TRPV1, and P2Y1 receptors were co-expressed in ~80% of small diameter DRG cells [54]. Furthermore, there was direct evidence supporting a functional relationship between P2Y1 and TRPV1 in DRG neurons; thus, Yousuf *et al*. demonstrated that ADP-induced activation of P2Y1 receptors facilitated capsaicin-induced currents, and this facilitatory effect was prevented by protein kinase C inhibition [55]. On the other hand, the relationship and interaction between P2Y1 and TRPV1 receptors have not yet been examined *in vivo*. In the current study, we examined the potential interaction between P2Y1 and TRPV1 receptors and demonstrated that this interaction resulted in the development of a TH response in ischemic rats. As indicated in Figure 7, we found that the creation of a more acidic environment in the ischemic hind paw resulted in the up-regulation of PKC-dependent pTRPV1 expression. Moreover, pre-injection of the P2Y1 receptor antagonist, MRS2179 significantly reduced this increase in pTRPV1 expression. Based on these results, we conclude that activation of P2Y1 receptors in an acidic environment modulates TRPV1 activity (i.e. phosphorylation at the S800 site) and ultimately causes the development of TH under peripheral ischemic conditions. Although we do not delineate the downstream individual molecular steps resulting from activation of P2Y1 receptors in this study, it is feasible to consider that activation of P2Y1 receptors can directly phosphorylate TRPV1 through Gq/G11 related intracellular cascades. When agonists stimulate this type of receptor, phosphatidylinositide-specific phospholipase C (PLC) is activated and subsequently causes hydrolysis of PIP2 in the plasma membrane. Inositol 1,4,5-triphosphate (IP_3_) and diacylglycerol (DAG) are generated by PIP2 hydrolysis resulting in the release of Ca^2+^ from intracellular stores and subsequent activation of the protein kinase C (PKC) pathway [56-58].

One of the questions resulting from the current study relates to explaining the mechanism by which acidic tissue conditions such as that associated with ischemia affects P2Y1Rs. One possibility is that the lower pH found in ischemic tissue changes the agonist affinity of the P2Y1 receptor. Many reports have demonstrated that extracellular protonation modulates the affinity of the ATP binding site and enhances the agonist potency of P2 receptors. For example, Li *et al*. demonstrated that extracellular protons regulated the function of P2X receptors by modulating the affinity of the ATP binding site [59]. Furthermore, extracellular protons have been shown to significantly potentiate the agonist potency of recombinant P2Y4 receptors, indicating the functional potentiation of P2Y receptors by protons [60]. Furthermore, we should also consider the possibility that the concentration of P2Y receptor agonists at the tissue site might be changed by lower tissue pH. In this regard, Dulla *et al*. reported that ATP hydrolysis to ADP and adenosine (by ecto-nucleotidases) was closely link to the PCO_2_ level and pH in hippocampal slices, and changes in nucleotides levels ultimately modulated neuronal excitability in the forebrain [61]. In addition, Sowa *et al*. demonstrated that prostatic acid phosphatase (PAP), which was expressed in nociceptive neurons and functions as an ectonucleotidase, had pH-dependent ectonucleotidase activity. At neutral pH, mPAP primarily dephosphorylated AMP; however, under acidic extracellular conditions, mPAP could dephosphorylate all purine nucleotides (AMP, ADP, ATP) [62]. Collectively, these reports suggest that the acidic condition of the tissue can change nucleotidase activity leading to increased dephosphorylation of ATP, which would result in an increase in ADP concentration and increased activatation of P2Y1 receptors.

## Conclusions

In conclusion, the present study clearly demonstrates that the presence of chronic ischemia in combination with increased tissue acidity can alter TRPV1 receptor sensitivity, not by a direct action of protons on the TRPV1 receptor, but rather by an indirect action of ATP on TRPV1 receptors that is mediated via a Gq-coupled P2Y1 receptor. Furthermore, this activation of P2Y1 receptors by an increase acidic tissue environment results in a PKC-dependent TRPV1 phosphorylation, which ultimately contributes to the development of thermal hyperalgesia. Thus, this study suggests a novel peripheral mechanism that may underlie the development of thermal hyperalgesia in chronic ischemic patients with severe acidosis.

## Competing interests

The authors declare that they have no competing interests.

## Authors’ contributions

SGK participated in the design of the study, carried out all of the experiments and wrote the manuscript. DHR and SYY guided the design of the study. JYM, SRC, HSC and SYK helped with analysis, interpretation of data and technical support. HJH, AJB, SBO and JHL assisted with the study design, interpretation of the data and the writing of the manuscript. All authors have read and approved the final manuscript.
